# Genome Sequence of *Arthrobacter globiformis B-2979* Phage *JanetJ*

**DOI:** 10.17912/micropub.biology.001351

**Published:** 2024-11-07

**Authors:** Arib Ahsan, Ruby S Crosthwait, Josephine J Crosthwait, Srilekha Davuluri, Omoye N Ehimare, Yihao Fan, Tiarra Nikitha Joseph Philomen Raju, Jamie Kim, Alexander Y Lee, Nicholas K Odani, Mugil V Shanmugam, Anmol Singhal, Alana J Snyder, Jessica W Sy, Grace Y Wang, George W Zhou, Christa T Bancroft

**Affiliations:** 1 Department of Biological Sciences, University of Southern California, Los Angeles, California, United States; 2 Department of Sociology, University of Southern California, Los Angeles, California, United States; 3 Department of Quantitative and Computational Biology, University of Southern California, Los Angeles, California, United States; 4 Department of Chemistry, University of Southern California, Los Angeles, California, United States; 5 Department of Gerontology, University of Southern California, Los Angeles, California, United States

## Abstract

Phage JanetJ was isolated on *Arthrobacter globiformis B-2979* and has siphovirus morphology. JanetJ's genome consists of 36,986 base pairs, encoding 52 putative protein-coding genes. JanetJ adds to the small number of previously isolated cluster FO phages, none of which encode identifiable immunity repressor or integrase functions, with the exception of phage Maja.

**Figure 1.**
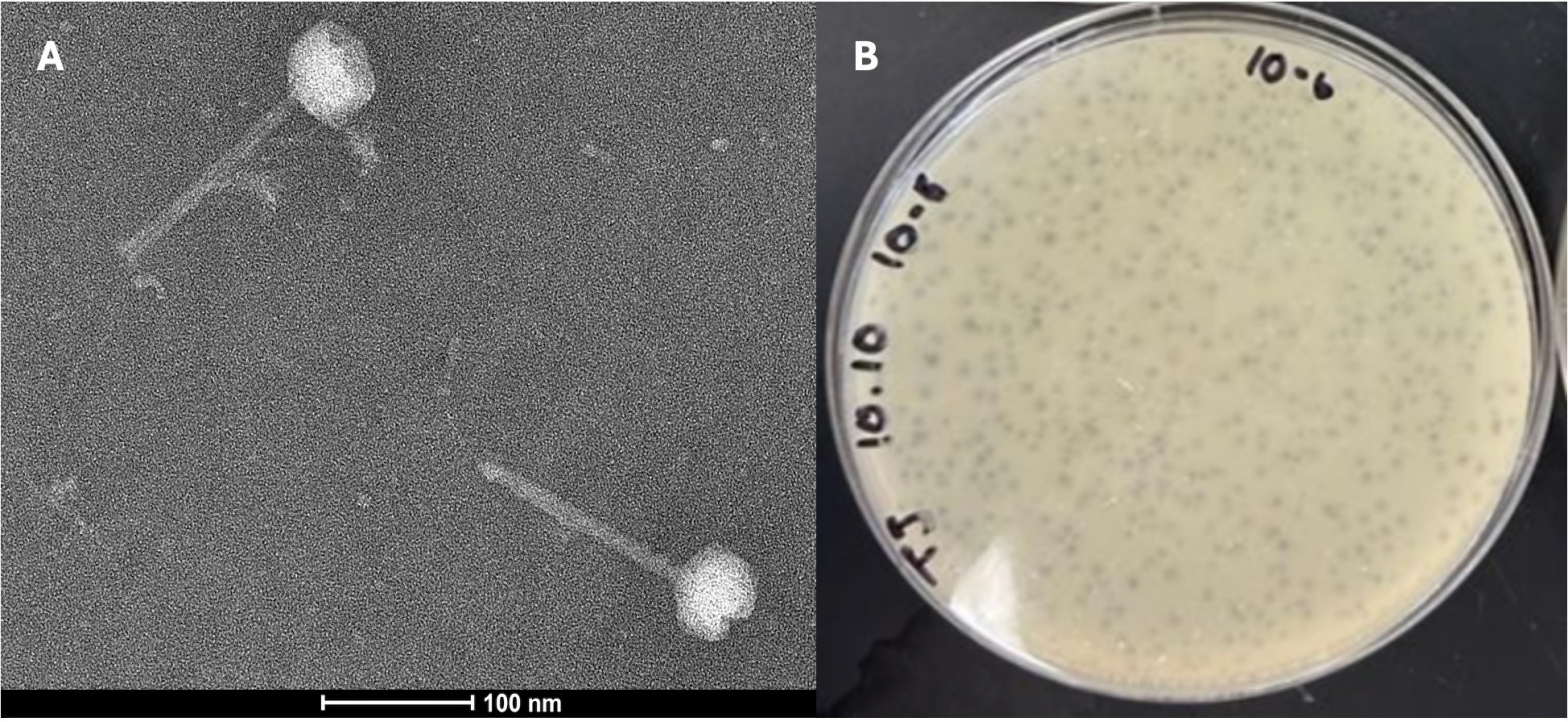
A) Nano-W negative stain (https://www.nanoprobes.com/products/Negative-Stains.html#nano-w) transmission electron micrograph (Talos F200CG2, 200KeV) (top left) and B) plaques for JanetJ. Scale bar is 100 nm.

## Description

As some of the simplest yet most diverse biological entities in the world, bacteriophages have gained relevance today in both clinical and ecological spheres of research (Hatfull 2022) . We aimed to discover phages that infect Arthrobacter, a genus that is host to a growing collection of phages and will permit population-level comparative genomics (Klyczek et al., 2017) . Phage JanetJ was isolated from a moist soil sample collected at the University of Southern California (34.019432 N, 118.285946 W) using standard procedures (Poxleitner et al., 2018) . Briefly, the soil sample was washed in peptone-yeast extract-calcium (PYCa) medium, the wash was filtered (0.22mm), the filtrate was inoculated with *Arthrobacter globiformis B-2979* and incubated with shaking at 30˚C for 48 h. The culture was refiltered, diluted and plated in soft agar containing *Arthrobacter globiformis B-2979* . After 24 h at 30˚C, JanetJ produced slightly cloudy, round, and uniform plaques, with a diameter of 1.35-1.5 mm (n=7) ( Figure 1 ). The phage were purified with 4 rounds of plating before being imaged by negative-stain (methylamine tungstate; Nano-W) transmission electron microscopy. JanetJ has siphovirus morphology, possessing a tail 145-147 nm in length and capsid of 54-56 nm in diameter (n=3) ( Figure 1 ).

Double-stranded DNA was purified using the Promega Wizard DNA cleanup kit, prepared for sequencing using the NEB Ultra II kit, and sequenced on an Illumina MiSeq (v3 reagents). Sequencing reads were assembled using Newbler v2.9 and checked for accuracy and genomic termini using Consed v29 (Gordon and Green 2013) , as described previously (Russell and Hatfull 2018) yielding a 36,986 bp genome with 3' single-stranded overhang (Table 1).

The genome sequence was automatically annotated using DNAMaster v5.23.6 (cobamide2.bio.pitt.edu) embedded with GeneMark v2.0 (Besemer and Borodovsky 2005) and Glimmer v3.02 (Delcher et al., 2007) . Following auto-annotation, Starterator (http://phages.wustl.edu/starterator/) was used to refine start sites. JanetJ encodes 52 putative protein-coding genes. No tRNAs were identified by Aragorn v1.2.38 (Laslett and Canback 2004) and tRNAscan-SE 2.0 (Lowe and Eddy 1997) . Default parameters were used for all software. Based on gene-content similarity of at least 35% to phages in the Actinobacteriophage database (Russell and Hatfull 2016) , JanetJ is assigned to phage cluster FO. HHPred (databases: PDB mmCIF70, Pfam-A, and NCBI Conserved Domain databases) (Söding et al., 2005) , NCBI BLAST (databases: nonredundant and Actinobacteriophage) (Altschul et al., 1990) , and Phamerator (database Actino_Draft) (Cresawn et al., 2011) were used to deduce the putative functions of proteins encoded by open reading frames. Of note, no immunity repressor or integrase functions could be identified, suggesting JanetJ is unlikely to establish lysogeny. This is in contrast to one other cluster FO phage, Maja, which encodes a putative immunity repressor and two integrase genes, though no experimental data for lysogeny has been reported for Maja. While Maja and JanetJ have 35% gene content similarity, the majority of gene conservation is within the first third of the genome encoding structure and assembly genes, whereas the remaining two-thirds, including the region encoding the lysogeny functions in Maja, is poorly conserved with JanetJ and other cluster FO phages. The genes in this latter region of cluster FO phages include those associated with DNA metabolism and many with unknown functions. Across all cluster FO phages, most of the genes are transcribed unidirectionally, with the exception of a handful of genes in each genome that are transcribed in the opposite direction, including those encoding the immunity repressor and one integrase in Maja and two putative helix-turn-helix DNA binding domain proteins in JanetJ.

**Table:** 

**Table 1. Sequencing Data and Genome Characteristics for JanetJ**	
Number of Sequencing Reads	426,273
Length of Sequencing Reads	150-base single-end
Coverage of Sequencing Reads	1728x
Genome Length (bp)	36,986
GC%	68.10%
Genome End Types	3' sticky overhangs(5' - TTCGCCTGGTA - 3')
Cluster Assignment	FO

**Nucleotide Sequence Accession and Read Numbers**

JanetJ is available at GenBank accession PP978789 and Sequence Read Archive (SRA) accession SRX24123888

## References

[R1] Altschul Stephen F., Gish Warren, Miller Webb, Myers Eugene W., Lipman David J. (1990). Basic local alignment search tool. Journal of Molecular Biology.

[R2] Besemer J., Borodovsky M. (2005). GeneMark: web software for gene finding in prokaryotes, eukaryotes and viruses. Nucleic Acids Research.

[R3] Cresawn Steven G, Bogel Matt, Day Nathan, Jacobs-Sera Deborah, Hendrix Roger W, Hatfull Graham F (2011). Phamerator: a bioinformatic tool for comparative bacteriophage genomics. BMC Bioinformatics.

[R4] Delcher Arthur L., Bratke Kirsten A., Powers Edwin C., Salzberg Steven L. (2007). Identifying bacterial genes and endosymbiont DNA with Glimmer. Bioinformatics.

[R5] Gordon D, Green P. 2013. Consed: a graphical editor for next-generation sequencing. Bioinformatics 29:2936–2937 10.1093/bioinformatics/btt515 PMC3810858 23995391

[R6] Hatfull Graham F. (2022). Mycobacteriophages: From Petri dish to patient. PLOS Pathogens.

[R7] Klyczek Karen K., Bonilla J. Alfred, Jacobs-Sera Deborah, Adair Tamarah L., Afram Patricia, Allen Katherine G., Archambault Megan L., Aziz Rahat M., Bagnasco Filippa G., Ball Sarah L., Barrett Natalie A., Benjamin Robert C., Blasi Christopher J., Borst Katherine, Braun Mary A., Broomell Haley, Brown Conner B., Brynell Zachary S., Bue Ashley B., Burke Sydney O., Casazza William, Cautela Julia A., Chen Kevin, Chimalakonda Nitish S., Chudoff Dylan, Connor Jade A., Cross Trevor S., Curtis Kyra N., Dahlke Jessica A., Deaton Bethany M., Degroote Sarah J., DeNigris Danielle M., DeRuff Katherine C., Dolan Milan, Dunbar David, Egan Marisa S., Evans Daniel R., Fahnestock Abby K., Farooq Amal, Finn Garrett, Fratus Christopher R., Gaffney Bobby L., Garlena Rebecca A., Garrigan Kelly E., Gibbon Bryan C., Goedde Michael A., Guerrero Bustamante Carlos A., Harrison Melinda, Hartwell Megan C., Heckman Emily L., Huang Jennifer, Hughes Lee E., Hyduchak Kathryn M., Jacob Aswathi E., Kaku Machika, Karstens Allen W., Kenna Margaret A., Khetarpal Susheel, King Rodney A., Kobokovich Amanda L., Kolev Hannah, Konde Sai A., Kriese Elizabeth, Lamey Morgan E., Lantz Carter N., Lapin Jonathan S., Lawson Temiloluwa O., Lee In Young, Lee Scott M., Lee-Soety Julia Y., Lehmann Emily M., London Shawn C., Lopez A. Javier, Lynch Kelly C., Mageeney Catherine M., Martynyuk Tetyana, Mathew Kevin J., Mavrich Travis N., McDaniel Christopher M., McDonald Hannah, McManus C. Joel, Medrano Jessica E., Mele Francis E., Menninger Jennifer E., Miller Sierra N., Minick Josephine E., Nabua Courtney T., Napoli Caroline K., Nkangabwa Martha, Oates Elizabeth A., Ott Cassandra T., Pellerino Sarah K., Pinamont William J., Pirnie Ross T., Pizzorno Marie C., Plautz Emilee J., Pope Welkin H., Pruett Katelyn M., Rickstrew Gabbi, Rimple Patrick A., Rinehart Claire A., Robinson Kayla M., Rose Victoria A., Russell Daniel A., Schick Amelia M., Schlossman Julia, Schneider Victoria M., Sells Chloe A., Sieker Jeremy W., Silva Morgan P., Silvi Marissa M., Simon Stephanie E., Staples Amanda K., Steed Isabelle L., Stowe Emily L., Stueven Noah A., Swartz Porter T., Sweet Emma A., Sweetman Abigail T., Tender Corrina, Terry Katrina, Thomas Chrystal, Thomas Daniel S., Thompson Allison R., Vanderveen Lorianna, Varma Rohan, Vaught Hannah L., Vo Quynh D., Vonberg Zachary T., Ware Vassie C., Warrad Yasmene M., Wathen Kaitlyn E., Weinstein Jonathan L., Wyper Jacqueline F., Yankauskas Jakob R., Zhang Christine, Hatfull Graham F. (2017). Tales of diversity: Genomic and morphological characteristics of forty-six Arthrobacter phages. PLOS ONE.

[R8] Laslett D. (2004). ARAGORN, a program to detect tRNA genes and tmRNA genes in nucleotide sequences. Nucleic Acids Research.

[R9] Lowe Todd M., Eddy Sean R. (1997). tRNAscan-SE: A Program for Improved Detection of Transfer RNA Genes in Genomic Sequence. Nucleic Acids Research.

[R10] Poxleitner M, Pope W, Jacobs-Sera D, Sivanathan V, Hatfull GF. 2018. HHMI SEA-PHAGES Phage Discovery Guide. https://seaphagesphagediscoveryguide. helpdocsonline.com/home

[R11] Russell Daniel A, Hatfull Graham F (2016). PhagesDB: the actinobacteriophage database. Bioinformatics.

[R12] Russell DA, Hatfull GF. 2018. Sequencing, assembling, and finishing complete bacteriophage genomes. Methods Mol Biol 1681:109–125. 10.1007/978-1-4939-7343-9_9 29134591

[R13] Soding J., Biegert A., Lupas A. N. (2005). The HHpred interactive server for protein homology detection and structure prediction. Nucleic Acids Research.

